# The Beneficial Effect of Human Amnion Mesenchymal Cells in Inhibition of Inflammation and Induction of Neuronal Repair in EAE Mice

**DOI:** 10.1155/2018/5083797

**Published:** 2018-06-24

**Authors:** Jun Shu, Xiaojuan He, Hong Li, Xue Liu, Xuemei Qiu, Tongliang Zhou, Ping Wang, Xiaojie Huang

**Affiliations:** ^1^Institute of Clinical Medical Science, China-Japan Friendship Hospital, Beijing, China; ^2^Institute of Basic Research in Clinical Medicine, China Academy of Chinese Medical Sciences, Beijing, China; ^3^School of Life Science and Engineering, Southwest Jiaotong University, Chengdu, China

## Abstract

Multiple sclerosis (MS) is a chronic inflammatory autoimmune disease of the central nervous system (CNS). Currently, there is still lack of curative treatment for MS. Mesenchymal stem cell- (MSC-) based therapy is recently the subject of intense interest in autoimmune diseases. Here, we investigated the therapeutic effect and potential mechanism of human amnion mesenchymal cells (hAMC) on inflammation and remyelination in experimental autoimmune encephalomyelitis (EAE) mice. C57BL/6 mice were immunized with myelin oligodendrocyte glycoprotein (MOG) 35–55 peptide. hAMC were injected intraperitoneal when EAE was successfully established. The results demonstrated that application of hAMC significantly ameliorated the disease severity and histopathological changes in EAE mice. The production of proinflammatory cytokines such as IFN-*γ*, TNF-*α*, IL-1*β*, and IL-17A in the spleen and CNS was dramatically inhibited. Moreover, CD4+ T cells and CD8+ T cells in the CNS were also significantly decreased in EAE mice after hAMC treatment. In addition, hAMC treatment also promoted the production of neuron-repair factors (NGF, CNTF, and BDNF) in the CNS of EAE mice. In conclusion, these results indicated that hAMC could attenuate the inflammation and promote the remyelination in EAE mice, which might be a promising cell source for the therapy of MS.

## 1. Introduction

Multiple sclerosis (MS) is the most common chronic inflammatory autoimmune disease of the central nervous system (CNS) in young adults. It is characterized by mononuclear cell inflammation, demyelination, extensive axonal damage, and axonal loss [1, 2]. Several lines of evidence indicated that aberrant autoreactive T cell responses, combined with dysfunction of the regulatory network of the immune system, played a central role in the pathogenesis of MS [3, 4]. Up to now, there is still no cure for MS. Current approved drugs predominantly exert their effects on the inflammatory disease process but hardly decrease neurodegeneration or promote CNS repair [5]. Therefore, looking for new treatment that can not only attenuate inflammation but also prevent neurodegeneration as well as support remyelination and repair of damaged tissue is urgently needed [6].

Mesenchymal stem cells (MSC) are considered as a readily available source for tissue engineering. They have multipotent differentiation capacity and can be differentiated into various cell types. Therefore, they are mainly used in organ transplantation and tissue repair in the past [7, 8]. Recently, due to their immunomodulatory properties, MSC have been regarded as promising therapeutic candidates for the treatment of autoimmune diseases such as insulin-dependent diabetes mellitus and rheumatoid arthritis [9].

Human amnion mesenchymal cells (hAMC) are isolated from the amniotic membrane of human placenta [10]. Compared with MSC from other sources, hAMC can be easily obtained with minimal ethical problem. More than 10^7^ hAMC could be isolated from one single amnion by simple enzyme digestion procedures [11]. More importantly, these cells do not express telomerase, which exclude the risk of tumor formation after cell transplantation [12]. Those properties make hAMC a new ideal MSC resource for clinical application. In our previous studies, we found that hAMC could decrease the production of proinflammatory cytokines, and further, they inhibited T lymphocyte proliferation not only in vitro but also in rats with collagen-induced arthritis, a classic animal model for human rheumatoid arthritis [13–15]. Besides having effect on immune cells and inflammatory cytokines, hAMC have also been proved to produce neuroprotective factors [16]. These evidences indicate that hAMC may have potential clinical use in the treatment of some CNS-related inflammatory diseases. Actually, using hAMC treating animal models with CNS diseases such as amyotrophic lateral sclerosis and spinal cord injury has been reported with success [17, 18]. However, whether it could be used for the treatment of MS has still not been explored.

Experimental autoimmune encephalomyelitis (EAE) is the most commonly used experimental model for MS. Many studies have successfully used this model to explore the immune and neural mechanisms and evaluate efficacy of potential therapeutic interventions in MS [19–21]. Therefore, in this study, we investigate the therapeutic effect and possible mechanism of hAMC in mice with EAE.

## 2. Materials and Methods

### 2.1. Isolation of hAMC

Placentas were obtained at elective cesarean section with informed consent. hAMC were isolated from abandoned human placentas according to our previous description [11]. In brief, amnion layer was mechanically peeled off from chorion layer and washed several times with Hanks' balanced salt solution (HBSS) without calcium and magnesium to remove blood. Then, the amnion was digested with 0.25% trypsin (Gibco BRL, Gaithersburg, MD, USA) at 37°C for 30 min. Further, the amnion was cut into pieces and digested with 0.1% collagenase V (Sigma-Aldrich, St. Louis, MO, USA) at 37°C for 30 min to obtain hAMC. Finally, separated hAMC were cultured in DMEM/F12 supplemented with 10% FBS (Gibco BRL, Gaithersburg, MD, USA). hAMC of no more than 3 passages were used for experiments. In addition, all the correlated ethic issues concerning this study were approved by review board of China-Japan Friendship Hospital.

### 2.2. Animals

Female C57BL/6 mice purchased from Beijing Vital River Laboratory Animal Technology Co. Ltd. (Beijing, China) weighted 18–20 g with 8–10 weeks of age. The mice were housed in a room with a temperature-, humidity-, and light-controlled environment. They were fed food and water *ad libitum* and allowed to acclimatize themselves for one week before the initiation of experiment. The study was approved by the Research Ethics Committee of China-Japan Friendship Hospital.

### 2.3. Induction of EAE

Primary progressive EAE model for C57BL/6 mice was established following the published protocol [22]. The mice were immunized subcutaneously on the back with 0.2 mL of myelin oligodendrocyte glycoprotein (MOG) 35–55 peptide (MEVGWYRSPFSRVVHLYRNGK, HPLC-purity: >95%) (ChinaPeptides, Shanghai, China) emulsified in CFA (Chondrex, Redmond, WA, USA) containing 4 mg/mL *Mycobacterium tuberculosis* H37Ra. These injections were distributed over the following three sites: one along the midline of the back between the shoulders and two on either side of the midline on the lower back. The final dose of MOG 35–55 and *Mycobacterium tuberculosis* H37Ra was 200 *μ*g and 400 *μ*g per mouse. Each mouse received an additional 400 ng of pertussis toxin (R&D Systems, MN, USA) by intraperitoneal injection of 200 *μ*L PBS on day 0 and day 2 postimmunization. Clinical scores were calculated blindly by two researchers daily according to a 0–5 scale as follows [23]: 1, limp tail or waddling gait with tail tonicity; 2, waddling gait with limp tail (ataxia); 2.5, ataxia with partial limb paralysis; 3, full paralysis of 1 limb; 3.5, full paralysis of one limb with partial paralysis of the second limb; 4, full paralysis of two limbs; 4.5, moribund; and 5, death.

### 2.4. Treatment

The treatment started from day 14 (at the disease onset) after primary immunization and lasted for 21 days. Mice were randomly divided into three groups: normal group, model group, and hAMC group. The mice in hAMC group were injected intraperitoneally with 100 *μ*L PBS containing 1 × 10^6^ hAMC. The mice in normal group and model group were injected intraperitoneally with the same volume of PBS.

### 2.5. Immunofluorescence Staining Assay

Isolated hAMC were seeded onto 24-well plates. After adherence, cells were washed with PBS and fixed with 4% paraformaldehyde for 20 min at room temperature. The fixative solution was removed and the cells were rinsed three times with PBS. The cells were processed with respective primary antibodies at 4°C overnight, including anti-CD105 antibody (mouse monoclonal, Invitrogen), anti-CD73 antibody (rabbit monoclonal, Abcam), and anti-vimentin antibody (mouse monoclonal, Santa Cruz). Then cells were incubated with corresponsive secondary antibodies conjugated to fluorescein (FITC, Jackson) or Alexa Fluor 488 (Jackson). Negative control was prepared by using isotype-controlled antibody. After washing three times with PBS, digital images were acquired with microscope.

### 2.6. ELISA

Blood of the mice was collected from the orbital artery and serum was isolated by centrifugation at 600 × g for 20 min. The levels of IL-1*β*, IL-17A, TNF-*α*, and IFN-*γ* in serum were detected by using Luminex Multi-factor Detection Technology (eBioscience ProcartaPlex) according to the manufacturer suggested protocol.

### 2.7. Histopathology

After mice were sacrificed, the spinal cords were quickly removed and postfixed with 10% neutral formalin for 48 h. Paraffin-embedded spinal cord cross-sections (5 mm thick) were dewaxed in xylol, rehydrated, and then stained with hematoxylin and eosin (H&E) and luxol fast blue (LFB) staining in order to detect tissue inflammation and demyelination, respectively. Histopathological examination was performed and scored in a blinded fashion as follows [24]: for inflammation: 0, no inflammatory cells; 1, a few scattered inflammatory cells; 2, organization of inflammatory infiltrates around blood vessels; and 3, extensive perivascular cuffing with extension into adjacent parenchyma, or parenchymal infiltration without obvious cuffing. For demyelination: 0, none; 1, rare foci; 2, a few areas of demyelination; and 3, large (confluent) areas of demyelination. Five serial sections of each spinal cord from each of eight mice per group were scored.

### 2.8. Immunohistochemistry

The cross-sections (5 mm thick) were dewaxed using xylene and dehydrated in a graded series of alcohols after incubation at 60°C for 1 h. The endogenous peroxidase activity was quenched with 3% H_2_O_2_, and heat-induced epitope retrieval was done in sodium citrate buffer. Sections were incubated with anti-CD3 antibody (Abcam, Cambridge, UK), anti-CD4 antibody (Abcam, Cambridge, UK), and anti-CD8 antibody (Abcam Cambridge, UK) overnight at 4°C, followed by incubation with SignalStain® Boost IHC Detection Reagent (HRP, Rabbit) (Cell Signaling Technology, Danvers, MA, USA) according to instructions from manufacturers. Final color product was developed with SignalStain DAB Substrate Kit (Cell Signaling Technology, Danvers, MA, USA), and then sections were counterstained with hematoxylin (Leagene, Beijing, China). Images were captured by LEICA DM6000B with a LEICA DFC300 FX (Leica Microsystems Ltd., Solms, Germany) at a magnification of 200x. Six fields were evaluated for each slide [25]. The numbers of positive cells per mm^2^ of spinal cord tissues were made by manual counting at Image-Pro Plus 6.0 software (Media Cybernetics, Rockville, MD, USA) [26].

### 2.9. Quantitative Real-Time PCR

IL-1*β*, IL-17A, TNF-*α*, and IFN-*γ* mRNA levels in the spinal cord were analyzed by quantitative real-time PCR. Total RNA was isolated from the spinal cord through tissue homogenate using TaKaRa MiniBEST Universal RNA Extraction Kit (TaKaRa, Kusatsu, Japan) according to the manufacturer's instructions. This procedure was done under RNase-free conditions. The total RNA (1 *μ*g) was reverse transcribed to cDNA using PrimeScript™ RT reagent Kit with gDNA Eraser (TaKaRa, Kusatsu, Japan) according to the instructions manual. The specific transcripts were quantified by quantitative real-time PCR using SYBR® Premix Ex Taq™ II (TliRNaseH Plus), ROX plus (TaKaRa, Kusatsu, Japan) and analyzed with ABI 7500 real-time PCR system (Applied Biosystems, Foster, CA, USA). Gene-specific primers were synthesized by Sangon Biotech (Shanghai, China), and the following primer sequences were used: CTCTCCACCTCAATGGACAGA (forward) and TGCTTGGGATCCACACTCTC (reverse) for IL-1*β*, CTCAACCGTTCCACGTCAC (forward) and ACACCCACCAGCATCTTCT (reverse) for IL-17A, ATGAACGCTACACACTGCATC (forward) and CCATCCTTTTGCCAGTTCCTC (reverse) for IFN-*γ*, GCCACAAGCAGGAATGAGAAG (forward) and GCCACAAGCAGGAATGAGAAG (reverse) for TNF-*α*, and TGGAGTCTACTGGCGTCTT (forward) and TGTCATATTTCTCGTGGTTCA (reverse) for GAPDH. The mRNA levels were normalized to GAPDH mRNA level. PCR was performed as 95°C for 30 sec, 40 cycles at 95°C for 5 sec and 60°C for 30 sec. The relative mRNA expression was calculated with comparative CT method.

### 2.10. Western Blot

The levels of NGF, CNTF, and BDNF in the CNS of EAE mice were detected by Western blot. Brain was collected and snapped frozen at −80°C immediately. Tissue homogenates were prepared in lysis buffer, consisting of 1 nM PMSF. Proteins were denatured, and equal amounts of proteins were electrophoresed in 12% bis-Tris/polyacrylamide gels and transferred to PVDF membranes. The membranes were blocked for 2 h in blocking solution and incubated overnight at 4°C with primary antibodies (Abcam, Cambridge, UK) diluted in blocking solution. Next, incubation with horseradish peroxidase-conjugated secondary antibody was performed at room temperature for 2 h, and immunoreactivity was detected by using enhanced chemiluminescence. Blots were scanned and analyzed for measurement of the band intensities with UN-SCAN-IT version 5.1 software.

### 2.11. Statistical Analysis

Statistical analysis was performed with SPSS 18.0 software. All data were expressed as mean ± SD. Differences in mean values of various groups were analyzed by ANOVA. Comparisons of numerical data between two groups were calculated by Student *t*-tests. Difference with *P* value < 0.05 was considered as statistically significant.

## 3. Results

### 3.1. Characterization of hAMC

**Figure 1:**
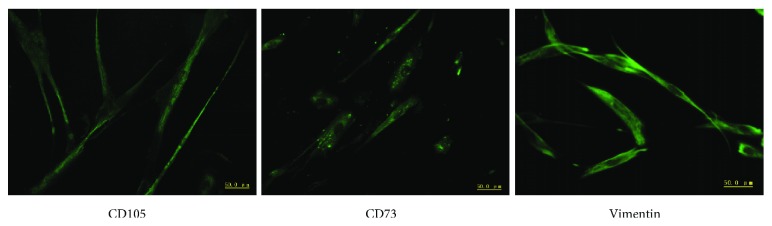
hAMC express stem cell specific markers. Stem cell markers—CD105, CD73, and vimentin—were shown by immunofluorescence in hAMC. Magnification: ×100.

Immunofluorescence staining assay showed that isolated hAMC expressed stem cell specific markers CD105, CD73, and vimentin (Figure 1).

### 3.2. hAMC Ameliorated the Symptom and Improved CNS Pathology of EAE Mice

**Figure 2:**
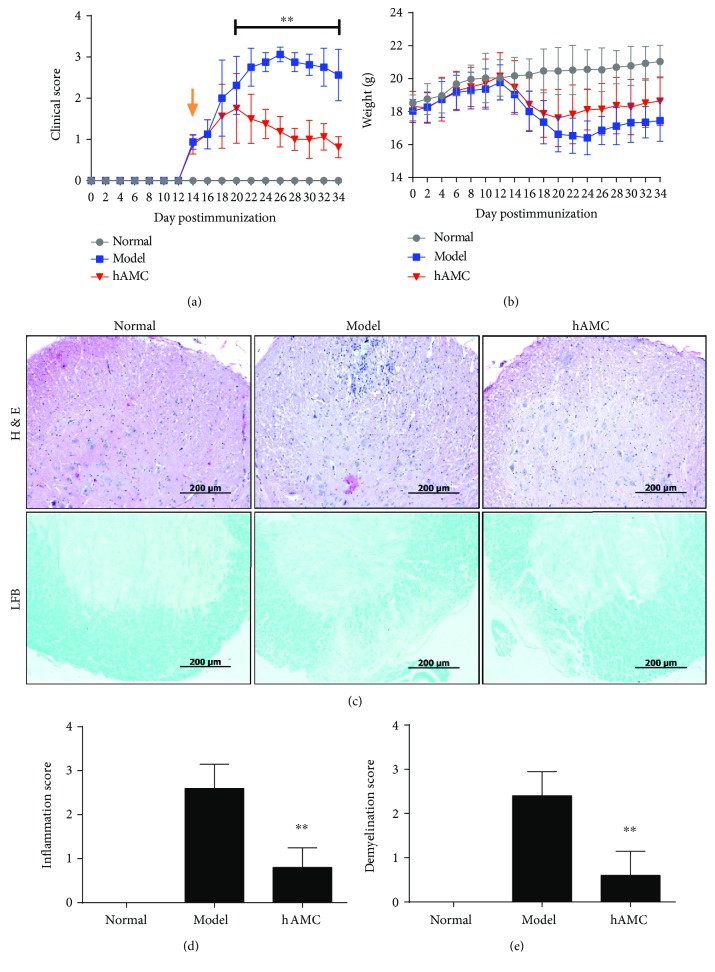
hAMC ameliorates the clinical symptoms and improves CNS pathology of EAE mice. (a) Time course changes of mean clinical score in the mice from respective group. Arrow shows the starting point of hAMC treatment. (b) Time course changes of body weight in the mice from respective group. (c) Representative histological finding of spinal cords from each group. Tissue sections from the spinal cords were stained with H&E or LFB, respectively. Scale bars, 200 *μ*m. (d) Histological inflammation score in each group. (e) Demyelination score in each group. Results are shown as mean ± SD. *n* = 8 mice per group. For pathological analysis, *n* = 5 sections per animal, eight mice in each group. ^∗∗^*P* < 0.01, compared to model group.

To determine the effect of hAMC on MS, we administrated hAMC in a classical MOG-induced EAE mice model. In our preliminary studies, we found that 1 × 10^6^ hAMC was optimal for suppressing EAE; therefore, this dose was chosen for the subsequent *in vivo* experiments. As shown in Figure 2(a), hAMC remarkably attenuated the clinical symptoms of EAE mice. The mean clinical score was obviously lower in hAMC group when compared to model group from day 20 till the end of the experiment. In addition, we found improvement of body weight in hAMC group to some extent, although there was no significant difference between model group and hAMC group (Figure 2(b)). In order to investigate the effect of hAMC on CNS pathology of EAE mice, we detected the inflammation and demyelination changes in the spinal cords by H&E staining and LFB staining, respectively. As shown in Figure 2(c), the model group showed significant vascular cuff-like changes and diffused inflammatory cell infiltration and demyelination compared with the normal group. Excitingly, hAMC treatment could improve the severity of these pathological changes. The inflammation score and demyelination score were obviously lower in the hAMC group compared to the model group (Figures 2(d) and 2(e)).

### 3.3. hAMC Suppressed Proinflammatory Cytokine Production in EAE Mice

**Figure 3:**
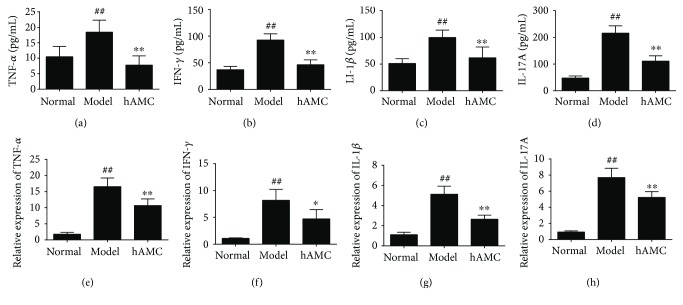
hAMC suppresses the production of proinflammatory cytokines in EAE mice. (a–d) Effect of hAMC on the production of cytokines in the serum. The levels of TNF-*α*, IFN-*γ*, IL-1*β*, and IL-17A in the serum of mice were determined by ELISA. (e–h) Effect of hAMC on the mRNA levels of cytokines. The mRNA levels of TNF-*α*, IFN-*γ*, IL-1*β*, and IL-17A in the spinal cords of mice were detected by real-time PCR. Data were mean ± SD. *n* = 8 mice per group. ^##^*P* < 0.01, compared to normal group. ^∗^*P* < 0.05, ^∗∗^*P* < 0.01, compared to model group.

To determine the anti-inflammatory properties of hAMC in EAE mice, we examined the levels of several important proinflammatory cytokines in serum and CNS of EAE mice. As shown in Figure 3(a)–3(d), production of TNF-*α*, IFN-*γ*, IL-1*β*, and IL-17A in the serum of EAE mice were significantly increased compared to the normal mice, whereas hAMC could remarkably decrease these proinflammatory cytokine levels. On the other hand, we also detected the levels of these cytokines in the CNS by real-time PCR. The results showed that compared to the model group, hAMC treatment could significantly lower the levels of these proinflammatory cytokines (Figures 3(e)–3(h)).

### 3.4. hAMC Decreased CD4+ T Cells and CD8+ T Cells in EAE Mice

**Figure 4:**
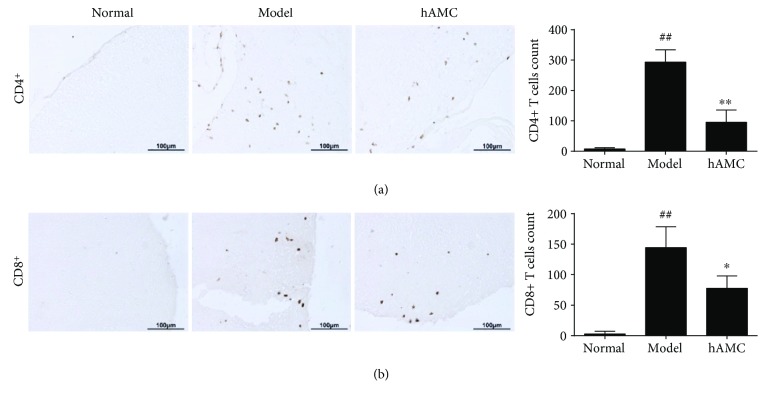
hAMC decreases CD4+ T cells and CD8+ T cells in the CNS of EAE mice. (a) The changes of CD4+ T cells in the spinal cords. (b) The changes of CD8+ T cells in the spinal cords. Left, representative images. Scale bars, 100 *μ*m. Right, the quantitative analysis. Six fields were evaluated for each slide. The numbers of positive cells per mm^2^ of the spinal cord tissues were made by manual counting at Image-Pro Plus 6.0 software. Data were mean ± SD. *n* = 8 mice per group. ^##^*P* < 0.01, compared to normal group. ^∗^*P* < 0.05, compared to model group, ^∗∗^*P* < 0.01, compared to model group.

To further investigate the anti-inflammatory mechanism of hAMC, we detected CD4+ T cells and CD8+ T cells in the CNS of EAE mice after hAMC treatment. Immunohistochemistry analysis showed that the numbers of CD4+ T cells and CD8+ T cells were remarkably increased in the spinal cords from the model group. hAMC treatment significantly reduced the numbers of CD4+ T cells and CD8+ T cells when compared with the model group (Figure 4).

### 3.5. hAMC Promoted Neuron-Repair Factor Production in EAE Mice

**Figure 5:**
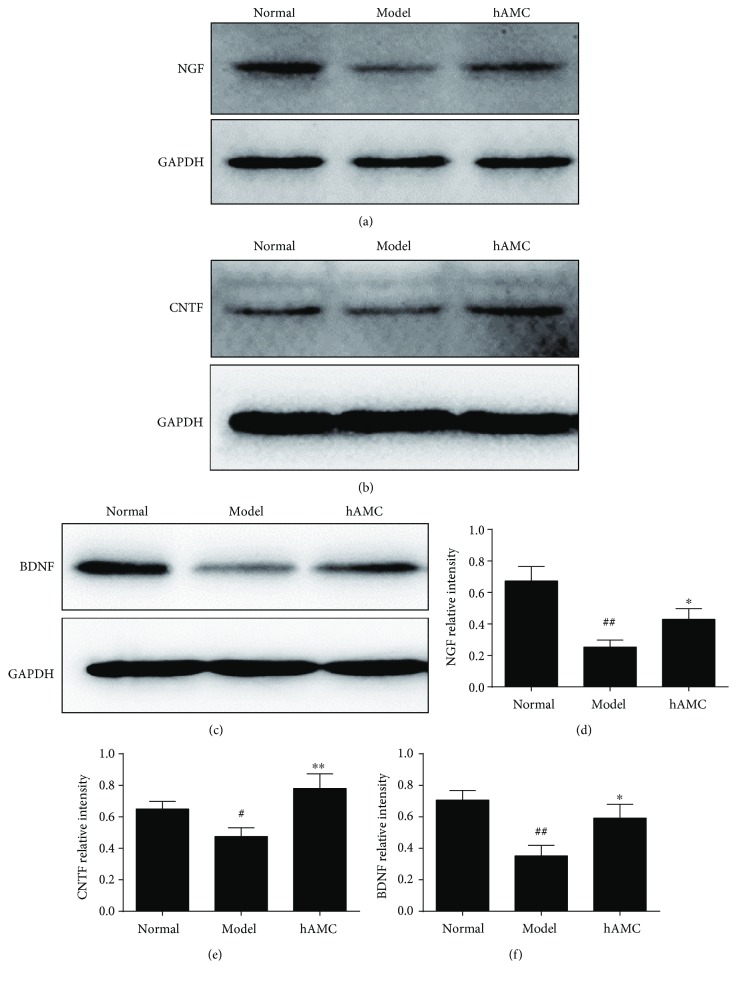
hAMC increases the neuron-repair factors in the CNS of EAE mice. (a–c) Representative images. (d–f) The quantitative analysis. The levels of NGF, CNTF, and BDNF in the brain were determined by Western blot. Data were mean ± SD. *n* = 8 mice per group. ^#^*P* < 0.05, ^##^*P* < 0.01, compared to normal group. ^∗^*P* < 0.05, ^∗∗^*P* < 0.01, compared to model group.

Previous LFB staining showed that hAMC treatment could lower the demyelination score in EAE mice, which implied hAMC might promote remyelination. To further investigate the action mechanism of hAMC, we next examined the levels of several important neuron-repair factors (NGF, CNTF, and BDNF) in the CNS of EAE mice after hAMC treatment. The results could be seen in Figure 5, which showed that hAMC treatment could significantly increase the expression of NGF, CNTF, and BDNF in the EAE mice.

## 4. Discussion

Recently, accumulating evidence showed that MSCs from different origins, including adipose-derived, bone marrow-derived, and umbilical cord-derived, could attenuate the disease progression in EAE animal models [27–29]. Furthermore, autologous bone marrow-derived MSCs transplantation and allogeneic umbilical cord-derived MSCs transplantation for the treatment of MS have been proved safe and effective in clinical trials, which showed that treatment improved the course of the disease, reduced the inflammatory response, and promoted neuroprotection [30–32]. Although MSCs provide a unique approach to the treatment of MS, how to obtain the abundant cells easily and how to avoid possible adverse effect in the clinic application are still rigorous challenges.

Human amniotic membrane is previously regarded as postlabor medical waste. However, accumulating evidences indicated it is a valuable biomaterial, because it possesses two distinct stem/progenitor cell populations: human amniotic mesenchymal cells (hAMC) and human amniotic epithelial cells (hAEC). hAMC are derived from embryonic mesoderm, while hAEC are derived from embryonic ectoderm. Both cells express stem cell marks including CD73 and CD105. The difference between the two types of cells is that hAMC expresses vimentin but not cytokeratin 19, whereas hAEC expresses cytokeratin 19 but not vimentin [33]. Despite both hAMC and hAEC display some similar properties including helping regeneration and repair of damaged tissues and organs [34], there still exist a few differences between these two kinds of cell types, such as immunoregulation [35]. As hAEC have already been reported to have immunosuppressive and therapeutic effect in MS, we wonder whether hAMC can also be used in the treatment of MS. Our study firstly demonstrated that hAMC could effectively attenuate the disease development in EAE mice. Because hAMC are easy to isolate and expand in vitro, at the same time, almost no risk of tumor formation, our study would help provide an alternative source of MSC in the MS treatment.

Although the cause and pathogenesis of MS are largely unknown, the current theories favor MS as an autoimmune inflammatory disorder of the CNS, wherein T lymphocytes play a central role [36]. Both CD4+ and CD8+ T cells have been demonstrated in MS lesions. On the one hand, a variety of stimuli including environmental factors, infection, inflammation, and autoimmune reactions induces autoreactive CD4+ T cells to be activated in the periphery. These activated CD4+ T cells adhere to the CNS endothelial and enter the CNS by transendothelial migration. After entering the CNS, these cells are reactivated by local and infiltrating activated antigen-presenting cells (APC), resulting in subsequent inflammatory processes and eventually in demyelination and axonal damage [37]. On the other hand, increasing evidence found that inflammatory infiltrates are often dominated by CD8+ T cells in the florid multiple sclerosis lesions [38]. These cells are probably much better suited to mediate CNS damage, since neurons, oligodendrocytes, and astrocytes in the CNS express MHC-I and therefore are preferentially recognized by CD8+ T cells [39]. In our study, we found that CD4+ T cells and CD8+ T cells were both increased in the CNS of EAE mice. These results were consistent with the previous reports. hAMC treatment effectively lowered the numbers of these cells, indicating that its therapeutic effect on EAE mice was at least partly through the decreased numbers of these cells.

Cytokine dysfunction responses are also observed in MS patients and EAE animal models [40, 41]. Overexpression of proinflammatory cytokines including IL-1*β*, IL-17, TNF-*α*, and IFN-*γ* has been shown to activate various inflammatory processes [29, 30]. Previous studies reported that these proinflammatory cytokines overexpressed in the blood, cerebrospinal fluid, and CNS lesions of MS patients [31]. Moreover, IL-1*β* deficiency significantly attenuated clinical symptom of EAE mice [27]. Therefore, regulating these overexpressed proinflammatory cytokines to physiological levels might be effective for the treatment of MS. In our study, we found that hAMC treatment effectively lowered the proinflammatory cytokines not only in the periphery but also in the CNS of EAE mice.

In MS, promoting remyelination is still a crucial therapeutic challenge [42, 43]. Stem cells on neural repair and neuroprotection function have been discovered and confirmed in recent years, as administration of these cells in experimental models of stroke and spinal cord injury obtained success [44, 45]. Studies indicated that stem cells had a neuroprotective activity because they could not only inhibit the production of inflammatory factors but also increase neurotrophic factors [46, 47]. However, whether hAMC can promote the neurotrophic factors production in EAE mice is still unknown. Neurotrophins, including NGF, CNTF, and BDNF, are a family of proteins that induce the survival, development, and function of neurons [48, 49]. They act by preventing the neuron from initiating programmed cell death, thus allowing the neurons to survive. They also induce differentiation of progenitor cells to form neurons [49]. Increasing evidence showed that the levels of these neurotrophins were significantly reduced in the CNS of MS patients and EAE mice and correlated with the deteriorated neuron damage, which implied that increasing the levels of these neurotrophins and/or maintaining their physiological levels in the CNS might be beneficial for MS [42, 50]. Actually, several related preparations, such as NGF eye drop and NGF infusion, have been approved in clinic to treat optic nerve injury, brain injury, etc. [51, 52]. In our study, we found that the levels of NGF, CNTF, and BDNF were significantly reduced in the CNS of EAE mice, which was consistent with the previous reports. Treatment with hAMC showed a significantly increase in the levels of these factors. These results indicated that besides anti-inflammatory effect, hAMC could also promote the neural repair in EAE mice.

Collectively, the results of the present study demonstrated that hAMC could effectively inhibit inflammation and promote remyelination in EAE mice, which was partly through decreasing the numbers of CD4+ T cells and CD8+ T cells, inhibiting the production of proinflammatory cytokines as well as increasing the levels of neuron-repair factors. Certainly, more studies still need to be done to further explain the mechanism of hAMC so as to promote its clinical application.

## Data Availability

The data used to support the findings of this study are available from the corresponding author upon request.

## References

[1] Thöne J., Linker R. (2016). Laquinimod in the treatment of multiple sclerosis: a review of the data so far. *Drug Design, Development and Therapy*.

[2] Rangachari M., Kuchroo V. K. (2013). Using EAE to better understand principles of immune function and autoimmune pathology. *Journal of Autoimmunity*.

[3] Zhang J., Weiner H. L., Hafler D. A. (1992). Autoreactive T cells in multiple sclerosis. *International Reviews of Immunology*.

[4] Hafler D. A. (2004). Multiple sclerosis. *The Journal of Clinical Investigation*.

[5] Wingerchuk D. M., Carter J. L. (2014). Multiple sclerosis: current and emerging disease-modifying therapies and treatment strategies. *Mayo Clinic Proceedings*.

[6] Van der Walt A., Butzkueven H., Kolbe S. (2010). Neuroprotection in multiple sclerosis: a therapeutic challenge for the next decade. *Pharmacology & Therapeutics*.

[7] Zhang L., Zhao Y. H., Guan Z., Ye J. S., de Isla N., Stoltz J. F. (2015). Application potential of mesenchymal stem cells derived from Wharton's jelly in liver tissue engineering. *Bio-medical Materials and Engineering*.

[8] Aziz Aly L. A., Menoufy H. E., Ragae A., Rashed L. A., Sabry D. (2012). Adipose stem cells as alternatives for bone marrow mesenchymal stem cells in oral ulcer healing. *International Journal of Stem Cells*.

[9] Lee H. K., Lim S. H., Chung I. S. (2014). Preclinical efficacy and mechanisms of mesenchymal stem cells in animal models of autoimmune diseases. *Immune Network*.

[10] Insausti C. L., Blanquer M., García-Hernández A. M., Castellanos G., Moraleda J. M. (2014). Amniotic membrane-derived stem cells: immunomodulatory properties and potential clinical application. *Stem Cells and Cloning: Advances and Applications*.

[11] Zhang K., Cai Z., Li Y. (2011). Utilization of human amniotic mesenchymal cells as feeder layers to sustain propagation of human embryonic stem cells in the undifferentiated state. *Cellular Reprogramming*.

[12] Bilic G., Zeisberger S. M., Mallik A. S., Zimmermann R., Zisch A. H. (2008). Comparative characterization of cultured human term amnion epithelial and mesenchymal stromal cells for application in cell therapy. *Cell Transplantation*.

[13] Shu J., He X., Zhang L., Li H., Wang P., Huang X. (2015). Human amnion mesenchymal cells inhibit lipopolysaccharide-induced TNF-*α* and IL-1*β* production in THP-1 cells. *Biological Research*.

[14] Shu J., Pan L., Huang X. (2015). Transplantation of human amnion mesenchymal cells attenuates the disease development in rats with collagen-induced arthritis. *Clinical and Experimental Rheumatology*.

[15] Shu J., Zhang K. H., Li H. (2012). Immunosuppression of human amniotic mesenchymal cells on allogeneic peripheral blood lymphocytes. *Zhonghua Zheng Xing Wai Ke Za Zhi*.

[16] Paradisi M., Alviano F., Pirondi S. (2014). Human mesenchymal stem cells produce bioactive neurotrophic factors: source, individual variability and differentiation issues. *International Journal of Immunopathology and Pharmacology*.

[17] Sun H., Hou Z., Yang H. (2014). Multiple systemic transplantations of human amniotic mesenchymal stem cells exert therapeutic effects in an ALS mouse model. *Cell and Tissue Research*.

[18] Zhou H. L., Zhang X. J., Zhang M. Y., Yan Z. J., Xu Z. M., Xu R. X. (2016). Transplantation of human amniotic mesenchymal stem cells promotes functional recovery in a rat model of traumatic spinal cord injury. *Neurochemical Research*.

[19] Constantinescu C. S., Farooqi N., O'Brien K., Gran B. (2011). Experimental autoimmune encephalomyelitis (EAE) as a model for multiple sclerosis (MS). *British Journal of Pharmacology*.

[20] Liu Y., Holdbrooks A. T., de Sarno P. (2014). Therapeutic efficacy of suppressing the Jak/STAT pathway in multiple models of experimental autoimmune encephalomyelitis. *Journal of Immunology*.

[21] Basler M., Mundt S., Muchamuel T. (2014). Inhibition of the immunoproteasome ameliorates experimental autoimmune encephalomyelitis. *EMBO Molecular Medicine*.

[22] Zhang Y., Li X., Ciric B. (2015). Therapeutic effect of baicalin on experimental autoimmune encephalomyelitis is mediated by SOCS3 regulatory pathway. *Scientific Reports*.

[23] Yang J., Jiang Z., Fitzgerald D. C. (2009). Adult neural stem cells expressing IL-10 confer potent immunomodulation and remyelination in experimental autoimmune encephalitis. *The Journal of Clinical Investigation*.

[24] Kan Q. C., Pan Q. X., Zhang X. J. (2015). Matrine ameliorates experimental autoimmune encephalomyelitis by modulating chemokines and their receptors. *Experimental and Molecular Pathology*.

[25] Guo Q., Zheng K., Fan D. (2017). Wu-Tou decoction in rheumatoid arthritis: integrating network pharmacology and in vivo pharmacological evaluation. *Frontiers in Pharmacology*.

[26] Soares R. M. G., Dias A. T., De Castro S. B. R. (2013). Optical neuritis induced by different concentrations of myelin oligodendrocyte glycoprotein presents different profiles of the inflammatory process. *Autoimmunity*.

[27] Liu R., Zhang Z., Lu Z. (2013). Human umbilical cord stem cells ameliorate experimental autoimmune encephalomyelitis by regulating immunoinflammation and remyelination. *Stem Cells and Development*.

[28] Shalaby S. M., Sabbah N. A., Saber T., Abdel Hamid R. A. (2016). Adipose-derived mesenchymal stem cells modulate the immune response in chronic experimental autoimmune encephalomyelitis model. *IUBMB Life*.

[29] Bai L., Lennon D. P., Eaton V. (2009). Human bone marrow-derived mesenchymal stem cells induce Th2-polarized immune response and promote endogenous repair in animal models of multiple sclerosis. *Glia*.

[30] Connick P., Kolappan M., Crawley C. (2012). Autologous mesenchymal stem cells for the treatment of secondary progressive multiple sclerosis: an open-label phase 2a proof-of-concept study. *Lancet Neurology*.

[31] Bonab M. M., Sahraian M. A., Aghsaie A. (2012). Autologous mesenchymal stem cell therapy in progressive multiple sclerosis: an open label study. *Current Stem Cell Research & Therapy*.

[32] Riordan N. H., Morales I., Fernández G. (2018). Clinical feasibility of umbilical cord tissue-derived mesenchymal stem cells in the treatment of multiple sclerosis. *Journal of Translational Medicine*.

[33] Wang J. P., Ouyang G. F. (2012). Phenotypic identification and differentiation potential analysis of two kinds of human amniotic cells. *Zhongguo Shi Yan Xue Ye Xue Za Zhi*.

[34] Manuelpillai U., Moodley Y., Borlongan C. V., Parolini O. (2011). Amniotic membrane and amniotic cells: potential therapeutic tools to combat tissue inflammation and fibrosis?. *Placenta*.

[35] Magatti M., Caruso M., de Munari S. (2015). Human amniotic membrane-derived mesenchymal and epithelial cells exert different effects on monocyte-derived dendritic cell differentiation and function. *Cell Transplantation*.

[36] Chitnis T. (2007). The role of CD4 T cells in the pathogenesis of multiple sclerosis. *International Review of Neurobiology*.

[37] Fletcher J. M., Lalor S. J., Sweeney C. M., Tubridy N., Mills K. H. G. (2010). T cells in multiple sclerosis and experimental autoimmune encephalomyelitis. *Clinical and Experimental Immunology*.

[38] Skulina C., Schmidt S., Dornmair K. (2004). Multiple sclerosis: brain-infiltrating CD8+ T cells persist as clonal expansions in the cerebrospinal fluid and blood. *Proceedings of the National Academy of Sciences of the United States of America*.

[39] Medana I. . M., Gallimore A., Oxenius A., Martinic M. . M. A., Wekerle H., Neumann H. (2000). MHC class I-restricted killing of neurons by virus-specific CD8+ T lymphocytes is effected through the Fas/FasL, but not the perforin pathway. *European Journal of Immunology*.

[40] Pasquali L., Lucchesi C., Pecori C. (2015). A clinical and laboratory study evaluating the profile of cytokine levels in relapsing remitting and secondary progressive multiple sclerosis. *Journal of Neuroimmunology*.

[41] Eng L. F., Ghirnikar R. S., Ling Lee Y. (1996). Inflammation in EAE: role of chemokine/cytokine expression by resident and infiltrating cells. *Neurochemical Research*.

[42] Modi K. K., Sendtner M., Pahan K. (2013). Up-regulation of ciliary neurotrophic factor in astrocytes by aspirin: implications for remyelination in multiple sclerosis. *The Journal of Biological Chemistry*.

[43] Pahan K. (2010). Neuroimmune pharmacological control of EAE. *Journal of Neuroimmune Pharmacology*.

[44] Li Y., Chen J., Chen X. G. (2002). Human marrow stromal cell therapy for stroke in rat: neurotrophins and functional recovery. *Neurology*.

[45] Akiyama Y., Radtke C., Honmou O., Kocsis J. D. (2002). Remyelination of the spinal cord following intravenous delivery of bone marrow cells. *Glia*.

[46] Zhou C., Zhang C., Chi S. (2009). Effects of human marrow stromal cells on activation of microglial cells and production of inflammatory factors induced by lipopolysaccharide. *Brain Research*.

[47] Okazaki T., Magaki T., Takeda M. (2008). Intravenous administration of bone marrow stromal cells increases survivin and Bcl-2 protein expression and improves sensorimotor function following ischemia in rats. *Neuroscience Letters*.

[48] Reichardt L. F. (2006). Neurotrophin-regulated signalling pathways. *Philosophical Transactions of the Royal Society of London. Series B, Biological Sciences*.

[49] Hempstead B. (2006). Dissecting the diverse actions of pro- and mature neurotrophins. *Current Alzheimer Research*.

[50] Chen X., Ma L., Jiang Y. (2012). Minocycline up-regulates the expression of brain-derived neurotrophic factor and nerve growth factor in experimental autoimmune encephalomyelitis. *European Journal of Pharmacology*.

[51] Lambiase A., Aloe L., Centofanti M. (2009). Experimental and clinical evidence of neuroprotection by nerve growth factor eye drops: implications for glaucoma. *Proceedings of the National Academy of Sciences of the United States of America*.

[52] Chiaretti A., Antonelli A., Genovese O. (2008). Intraventricular nerve growth factor infusion improves cerebral blood flow and stimulates doublecortin expression in two infants with hypoxic-ischemic brain injury. *Neurological Research*.

